# Teenagers and Playing: Are Pastimes Like Neknominate a Usual Response to Adolescence?

**DOI:** 10.3390/children1030339

**Published:** 2014-10-22

**Authors:** Perry Else

**Affiliations:** 1 Sheffield Hallam University, Sheffield S1 1WB, U.K.; 2 International Play Association—England, Wales and Northern Ireland (IPA-EWNI), c/o 70 Kilmorie Rd, Forest Hill, London SE23 2ST, U.K.

**Keywords:** benefits of play, play, risk, children and young people’s health, obesity, teenagers, adolescence, integral play framework, brain growth, culture, not-play, play deprivation, technology and play

## Abstract

While “outside of society” for much of the last sixty years, adolescents have attracted attention in recent times because of perceptions of their anti-social and, in some cases, violent behaviour. Teenagers face many challenges on their journey to adulthood; growth spurts, hormone developments and changes in the structure of the brain. These biological challenges have been affected since around 1990 by the impact of technology and the subsequent cultural changes. Activities, like the technology-driven, socially-networked pastime, Neknomination, amongst others, meet basic drives that gym-based activities do not. Adults are increasingly concerned about unhealthy patterns of behaviour that suggest that this coming generation of adults will not live as long as their parents, causing misery and putting additional economic pressures on families and society if the expected standards of living and health are to be maintained. The pressures facing teenagers are many, but a concerted effort by adults to change their attitudes towards children and young people to help rather than instruct may assist with meeting their needs and those of society.

## 1. Background: Healthy Development of Children and Young People

Changes in society and human environments over the last thirty years have been faster than at any time in recorded history, yet are no less profound for that. Almost every aspect that impacts on a human’s life has changed in the post-modern world: domestic and global polices, social cohesion, protection of personal and property rights and invested wealth, investments in research and development and infrastructure, the development of human capital through education, healthcare and child care and the protection and stewardship of natural capital [1].

While these issues may seem remote to children and young people, they will have profound impacts on the support and choices available to them, and it is recognised that the context of a person’s life is as important as the environment and physical resources supporting that person [2]. Most children are born with a wide range of abilities that will become manifest if given the appropriate type of experience and support, yet different environments will profoundly affect those life chances.

When looking at healthy development for children and young people, there is often a focus on physical development and the correlating avoidance of obesity [3]. The engagement of young people in physical activity programmes has gained prominence, as adults are increasingly concerned about unhealthy patterns of teenage behaviour that suggest that this coming generation of adults will not live as long as their parents [4]. The adult solution around the world has been shown to be the provision of organised sports and activity sessions aimed at lowering obesity levels and establishing a life-long habit to exercise.

Regarding physical health and obesity, research carried out in the U.K. in 2008–2009 [5] revealed that primary school children were sedentary for over six hours a day, with girls being particularly inactive (nearly four out of ten). Similar figures were reported in 2010–2011 with children getting heavier, especially in urban and deprived areas [6].

Reports on health tend to focus on the cost of programmes e.g., obesity in the U.K. costing over £5 bn per year [7], yet as will be shown, it is motivation and quality of life that ultimately will change young people’s behaviour. The guidance offered by the U.K.’s National Health Service, while well intentioned, may not be the answer needed, as it focussed on gym-based activities, such as sit-ups, push-ups, gymnastics, resistance exercises, weight machines, rock climbing and sports, such as football, basketball and tennis [8]. Twelve- to sixteen-year-olds do not stop playing through lack of knowledge about fitness and obesity; more information about diet and exercise is not needed. Games, like Neknomination (see below), meet basic drives that gym-based activities do not. Adults and policy makers should understand more of the pressures on and desires of young people in order to help support and help change activities and behaviours.

Some understanding is emerging; research in Australia showed that young women aged 12–16 years equated dissatisfaction with their body image to the perception of poorer health and related health behaviours, such as low physical activity levels, dieting and external physical activity motivators [9]. The research team concluded that adult-led interventions were needed to promote the young women’s positive body image and increase intrinsic motivation for physical activity before physical activity levels would rise among adolescent girls in the cohort. “Intrinsic motivation—the drive to do something because it is interesting, challenging and absorbing” [10] is necessary if individuals are to feel motivated to engage in new activity or change their behaviour; targets set by others—extrinsic motivators unless linked to collective benefits—are increasingly failing in the modern world.

Another example from the U.K., the Women’s Sport and Fitness Foundation (WSFF) [11], looked at the views held by young women and men about physical activity, sport and PE (physical education) lessons. They reported that only 12 percent of young women aged 14 get enough physical activity each week; roughly half that of young men at the same age. The WSFF found that young women want to be active, take part in physical activity and remain healthy, but the researchers attribute the lack of participation to young women feeling they do not have a suitable outlet for their activity. In earlier times, young people would have been busy with adult-directed activities at home or in school, yet in the twenty-first century, they feel it essential to fit physical activities into their cultural framework.

Finally, a study from Denmark [12] revealed that there was no relationship between families’ socio-economic position and the amounts of general physical activity in children and young people; though organised sports showed an increase in young peoples’ activity, which was attributed to the economic and social capitals held by the family. Therefore, when activities are supported and paid for by parents, adolescents will take part, but their own self-chosen activities are hardly different from the rest of the young population; other pressures must be at work.

## 2. Playful Activity Is Good for All Aspects of Being Human

All humans, but children and young people especially, experience life holistically and with integrated experiences; it is not possible to isolate physical actions from the space in which they occur. The growth in social media amongst young people shows that the desire to link with others is vital, yet the thousands of messages sent every day are about personal activities, what is happening, what was done, what excites them. The integral four quadrant model [13] demonstrates how the primary activities of humans link together to satisfy most human desires and needs. The model has been adapted and amended as the Integral Play Framework [14] to link to play activities for children and young people.

**Figure 1:**
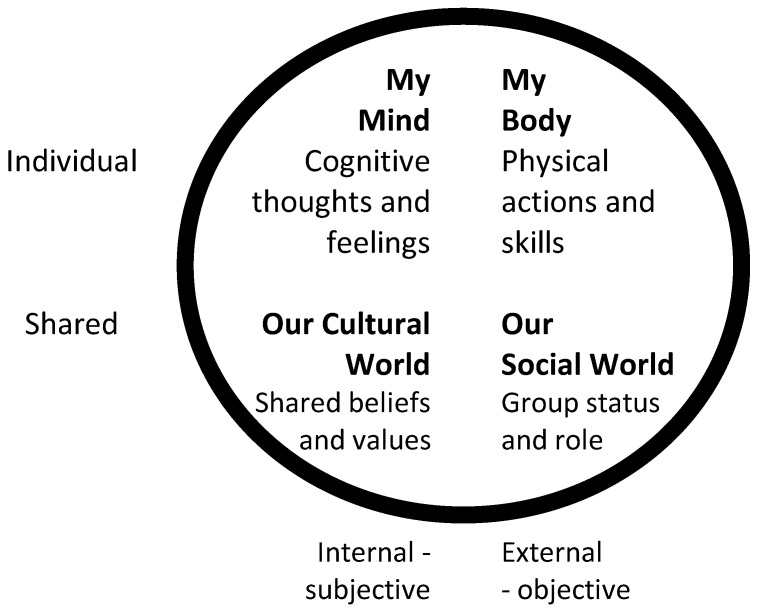
The Integral Play Framework.

The balance of physical activities with cultural interests brings together the objective reality of exercise and bodies in space with the subjective opinions of those “operating” the bodies; humans are not just robots carrying out mechanical motions, nor are they cultural identities remote from the world. The actions of the body impact on the feelings of the person and the feelings change the way the body is used/operated. When living in the world, humans also use their cognition to make decisions about the world and, when interacting with others, will be aware of their status in that social framework, whether they are a leader of the group, a partner or a follower. These four ways of interacting with others and the ecology are described by the Integral Play Framework [15]; see Figure 1.

The circle represents the individual young person and the four quadrants within that circle the four main ways that humans interact with others; two “internal” and mind based, two “external” in the physical realm, yet all interacting and affecting the others. Using the example of physical activity, sport and PE lessons, it may be seen that exercising the body is only part of the motivations for undertaking such a task. If there is a group status or role to be gained from the activity, then the activity may be undertaken in order to fit in or form good links with others; or the activity may be carried out if there are enough cognitive or emotional arguments to motivate the person—e.g., feeling good as a result of exercise or understanding that a limit on calorific intake may affect body shape and weight. Finally, a shared culture could influence the type and range of activities an individual undertakes with others; a strong motivator, especially for adolescents. Each of these quadrants could be a prime motivator or actions could be a combination of two of more of the quadrants. The framework describes the actions and choices of individuals, yet those choices may be affected and influenced by others in the environment or the opportunities in the environment itself. This in part explains why Neknomination was a more popular craze than gym-based activities for a time with some young people.

A human being is a time-sample of human nature. The whole person is physical if viewed from one angle; psychological if viewed from another. There are the soma (the body of an organism) and psyche (the human mind, conscious, and unconscious) [16].

## 3. Cultural Frameworks

Young people are very aware of contemporary culture, the effect it has on them and the impact they have through cultural behaviours, such as social media, socialising and rebelling, whether overt or passive, against the dominant traditions. The complexity of cultural transmission and the resultant changes in society were explained by Konner: “The issue with cultural evolution is that it is quite different from biological evolution, and one of its meanings is the succession from hunting and gathering to horticulture and/or pastoralism, to full-scale agriculture, to industrial and post-industrial modes of subsistence—a succession that entails changes in social and political complexity... Transmitting culture to children and young people is more complex and much less well understood than genetic inheritance” [17]. The early stages of this continuum would have required young people to learn the rules of the culture in order to survive; for example, a lack of understanding of food acquisition would eventually lead to death. Yet, in many societies in the minority world over recent years, food has been unreservedly available, leaving young humans to create their own cultures and belief systems. As Edelman put it, “Consciousness is not a thing; it is a process”; human minds are not determined by genetics, but by the interaction of chosen activities with the environments and opportunities available [18]. Genetics alone do not determine a human’s behaviour; culture plays a major part in shaping an individual’s behaviour, beliefs and attitudes.

## 4. Brain Growth and the Playful Development of Consciousness

There is now strong evidence that one reason for the evolution of playing is to help develop more efficient brains by enhancing cortical connections; that when humans play, the nerve signals that bodies generate create pathways in the brain that help with brain development [18,19]. The brain expands in volume four times from birth to the teenage years, and the brain structure and function in adults is a product of both evolution and individual development [20]. Research has shown that the first eight years of a human’s life are critical for facilitating brain development, with ages from birth to three years being especially crucial. The more children play in this period, the more connections are made between the various parts of the brain and especially through the corpus callosum, the band of cells that connect the left and right sides of the brain and that help integrate what are often two completely different views of the world. When children play cognitively, they develop mental wellbeing, brain elasticity, autonomy, as well as resilience [21]. Yet, a second critical phase of brain growth emerges when humans enter adolescence.

There is evidence that the cerebellum continues to change throughout adolescence; it is one of the last structures of the brain to develop in adulthood. It still needs to experience flexibility and stimulation. The way to make a better brain is not through hours of homework and training, what the brain wants is play; it grows best when it is allowed to play [22]. Smith quotes Panksepp as saying, “Play is quintessentially capable of activating the very best that the (brain) is capable of” [23]. Additionally, as brain functions increase, the variety and complexity of play increases; is it any wonder that the animals with the largest brains are also amongst some of the most playful? [19].

The brain remains flexible during pubescence and adolescence and only as adulthood approaches does it start to fully consolidate its structure (through myelination). If the brain were totally organised and set in its structure too early, human beings would never be able to learn the things they need to survive [24]. Myelination is a process of insulating neuron cell bodies, keeping the brain’s electrical signals on their intended paths and increasing their speed. This process in the developing teenage brain has good and bad consequences. After neurons are completely myelinated, they are more efficient and twice as fast, a desirable trait in adults who may need to respond quickly to challenges in the environment, but the brain cells also become rigid and less flexible. This is why, for instance, an adult reports finding it harder to learn new languages or new skills, hence the proverb about teaching old dogs new tricks. Adults can learn facts faster than children, they understand the methods and have models to assimilate new ideas into; however, the related skills of word pronunciation or understanding of sounds is much harder to grasp, and lack of confidence prevents most adults revealing their deficit to others, a reluctance that most children and many teenagers do not experience in certain contexts. There are many changes during adolescence that teenagers go through and help them enter a phase of risk taking and exploration that fades as they reach adulthood.

## 5. Teenagers Are Naturally Changing

There are key changes that young people go through, starting on average at 12 for girls and 14 for boys. Anecdotal and media claims state that these ages are dropping, yet research supports the traditional ages and suggests that cultural appearances are what explain the impression of lower ages for maturity and one or two highly reported media stories; most young women still wait for physical maturity and security before starting a family [25]. Evolutionary changes do not take place in one generation, but over a much longer period.

The physical changes during adolescence include growth spurts of 200–300 mm over a period of two or three years; increasing weight, height, heart size, lung capacity, muscular strength and giving teenagers ravenous appetites. Bone growth in adolescence is faster than muscle development, which can result in their lack of coordination and clumsiness. Puberty can be revealed by voice changes, emergence of body hair, growth of internal and external reproductive organs, production of female and male hormones and acne. Sex characteristics show in young women with breasts enlarging and menstruation beginning and erection and ejaculation for young men [26].

During the teenage years, loyalty for many shifts from parents to peers, who become sources for standards and models of behaviour, influenced in recent years by media advertising. Adolescents copy and display fads of extremes in clothing, speech and mannerisms, which have cycled faster and faster since the middle of the twentieth century. Young adolescents (as their parents before them did) believe their experiences are unique and dramatic. This is demonstrated in the adolescent’s search for independence, which frequently results in conflict with anyone in an authoritative positions [26].

Mentally, cognitively, the adolescent is able to participate in abstract thinking (Piaget’s formal operational stage), enabling them to process and group ideas; adolescence also brings egocentrism, where individuals believe everything centres on their appearance, thoughts and behaviours, and that critical thinking emerges from taking a firm position and arguing their point of view. Teenagers are curious and exhibit a strong willingness to learn things they consider useful, adding to the belief that they are invincible and incapable of experiencing anything harmful, leading to engagement in risk-taking or reckless behaviours [26].

Teenagers and their need for diversity and new experiences are also models for Gardner’s theory of multiple intelligences, which argues that logico-mathematical and linguistic intelligences, though valued in most modern societies, are not the only forms of intelligence. Varied forms of intelligence that are clear within teenagers and children (and some adults) are bodily-kinaesthetic, spatial, naturalist, musical and moral, amongst others [27]. Certainly, much teenage behaviour may have its evolutionary roots in the basic drive to mate, rather than a desire to fit into the social order or traditional norms [24].

## 6. Risk Taking in the Econiche

As well as the influences of others and genes on the teenager, the impact of the environment has a significant sway on growing adolescents. Research on rats’ brains revealed that a complex environment with lots of stimulus made the rats’ brains grow and change; toy-playing rats were also shown to be quicker at complex tasks [28].

Edelman [18] made a similar statement when talking about the environment that children grow and play in: “The econiche in which animals must survive has an enormous number of signals to which an individual must adapt”. In the modern world, human brains still need these signals in order to adjust to the planet; there is a danger that as environments are increasingly controlled and “made safe” for humans (especially in the industrialized world), the necessary richness and diversity are lost and so is the teenagers’ ability to develop and adapt.

Fortunately, it has been shown that play contributes to coping with stress, resilience and problem solving [19,29]. Connecting with others, learning the rules and developing physical skills all help most animals fit into their “gang” and become skilled at the incidents that life throws at them. Playing in challenging environments also aids recovery when stress hormones are released to help animals change or evade challenges they face; animals not used to such exposure take longer to calm down and so remain stressed for longer [19].

In humans, the confidence learned and developed though experience aids the emergence of positive emotions, which can help with both physical and psychological health issues by promoting resilience, endurance and optimism [30].

## 7.Neknominate and Other Modern Risks

Risk taking is a normal tool of development, and teenagers often define their identity through risk [31]. However, there is a threat that modern worlds, while still varied in their stimuli, offer reduced access to developing humans, who choose not to expose themselves to the chances on offer, but follow fads and crazes. As Strauch put it, “Modern rebels are lazy… There are too few outlets for normal risk-taking these days; the paths toward success are too limited” [24].

Such limits lead in part to the rise of social network games, such as Neknominate, the drinking game that started in Australia before spreading around the touch-screen, mobile-device carrying world. The game involves people (often teenagers) filming themselves drinking excessive amounts of alcohol (prohibited for those under 18 and under 21 in various countries), often while doing outlandish activities before posting images on friends’ sites and then nominating someone else to continue with another activity or drink. In the U.K., Neknominate has been linked to several deaths, prompting calls for social networking sites, such as Facebook and Twitter, to introduce warnings in an attempt to curtail or stop the activity [32]. In spite of that advice, the behaviour will only change when participants see value in other activities that meet their needs and desires. If adults are to work with teenagers in supporting healthier behaviour, they will need to consider the motivations and pressures on young people at the current time.

Other forms of play that adolescents have participated in since the millennium include extreme forms of the risky children’s game “chicken” (a form of deep play), such as street running or parkour, tomb-stoning, jumping into the unknown, buildering or building climbing, base jumping, bungee jumping, extreme ironing, off-piste snowboarding and skiing and modern skydiving, skimming just meters above the ground, sometimes with fatal consequences. Media reports of these activities also reveal that many of them add further challenges to social norms by being carried out naked, with no safety equipment, in ultimate conditions or even while participants get married. Many of these activities are intense forms of deep play [33] that allow young people to encounter risky or even potentially life-threatening experiences, testing the participants’ physical and mental reserves, while aiming to be satisfying, pleasurable and thrilling, with a deep sense of being alive.

## 8. So What Is Play?

There are many definitions of play, but a recent meta-analysis of authors’ views [15] suggests that play must be a self-chosen, engaging and satisfying activity or the player will stop playing. The pleasure of play has been recognised as contributing to many things; feeling good helps with “flexible thinking and problem solving, mastery and optimism” [34].

While play is self-chosen, nonetheless play is never fully free, as some definitions claim—children and young people cannot really fly; they can never get everyone to play “my” game, and so on. For play to be self-chosen, the player has to feel sufficiently safe, physically and psychologically, *i.e.*, not threatened excessively by others or the environment [21]; though certainly, there are exceptions, hence the “sufficiently”. For example, locomotor play, movement in all directions, will be different for a toddler and a teenager; the skill needed, the type of activity and the point of the play will vary across the ages and between individuals; and of course, older children may be socialising and problem-solving while running around. In some parts of the U.K. (and with similar activities across the world), children still do door-knocking, usually around Halloween. This is a fairly harmless activity of knocking on a series of doors and then running away before the occupants catch you; a bit of fun and no one should get harmed, though it is likely these days that they do not knock on the doors showing pictures of dangerous dogs and the legend “I live here” on the front door. Therefore, play is self-chosen with willing participation from the player.

Active engagement is necessary to sustain the play, responding to feedback from the environment and from other players: Are the tree branches still strong enough to hold me if I go higher? What role do I have in this game; do we need to change it? Playful engagement often leads to players losing track of time as they become immersed in their activity, which is usually a whole mind and body experience, with the player getting stimulation and satisfaction throughout while the activity still has the opportunity for newness or new experiences. The love of new things is called neophilia: Pink explained it thus: “Our basic nature is to be curious and self-directed”—and if as adults we are, “passive and inert, that’s not because it’s our nature. It’s because something flipped our default setting” [35]. Play is often described as “fun”, though five minutes watching children playing helps us realise that fun does not describe fully the rewards of play for them. Playing may be fun, but it can also be pleasurable, rewarding, satisfying, done for its own sake to experience the crossing of a boundary, done for the exuberance of it just to experience a feeling (jumping in a river, breaking rules, saying “no”); it is much more than just “fun”.

Playful activity is important for social, physical and intellectual development, but also for another theme, which may be the most important from an evolutionary perspective—cultural development. Playful activity, while benefitting the individual, is usually a group activity, where the rules and activities are defined by those playing—how far to roam, what the rules are to be “on”, how to move (often with self-limiting motions), how to get free, and so on. Many games involve movement whether using large muscle groups for running and climbing or fine movement, such as collecting, weaving or pattern-making; while moving around children and young people will be developing tactics to win, stay with their friends, outwit the chaser; this is the beginning of cognitive or intellectual development, alongside the symbolic recognition that leads to language development. Most playful activity involves leaders and followers, whether “real” or “pretend”; through play, children learn about social rules and who is in charge, who’ is fair, who picks on you when no one is looking. Additionally, children and young people learn about their culture, the dominant beliefs and values, how to behave in the street and school, but also how to behave in the mosque and minster. Of course, when children grow older, they will learn the games that are not allowed in certain cultures; for a time, they will comply, though the braver ones will begin to play with the systems and ask “why?” Teenagers will search for independence, exploring the rules in their immediate econiche and challenging those in positions of authority. Some adults will try to constrain this activity, describing it as anti-social, or try to teach the “right way” to behave through play.

As Grieshaber and McArdle stated, “The trouble with the idea that play is children’s natural way of learning is that ideas about what is natural in children are selective. They are a conglomeration of science, tradition, history, culture, and other ideas. And they vary across time and place.” [36]. Some modes of play will be affected by what is happening in those worlds; when a child states to a playmate, “I know the rules—I am a Muslim/I am a Catholic, *etc.*,” is that a fully conscious thought or a learned reaction from adults? Adolescents will begin to challenge these attitudes in their journey to independence, and this will bring them into conflict with the grownups. While very exceptional, think of Ms. Malala Yousafzai, the Pakistani student who was shot by the Taliban after speaking out about girls’ education. Fortunately, most teenagers do not endure such dire consequences when they start saying “no” to adults. Naturally, not everything that young people do on their way to adulthood may be considered play or playful, yet playful approaches still have their benefits.

The evidence is clear that play has a role in improving physical and social health, psychological wellbeing, creativity and divergent thinking, attention and cognitive functioning, child development and educational achievement and conduct [37]. Yet, not all young people access play on a regular basis.

## 9. The Not-Play Function of Play Type Activities

With the growing global trend to reduce obesity, agencies and settings are introducing what they consider to be playful sports and games with the stated outcome of burning up calories and reducing weight. The prompt for this type of programme is supported by research, such as that by Dehghan *et al.*: “It is more difficult to reduce excessive weight in adolescents and adults once it becomes established; therefore, it may be helpful to initiate obesity prevention interventions during early childhood” [38]. On the other hand, these programmes may not be playful, involving as they do adult-led activities conforming to adult-planned curricula, which, in part, explains why children and young people become frustrated with them and give them up when they can.

As playful activity often “wastes energy” or time, exposes the playful to noisy, aberrant behaviour and displays weakness until mastery is gained, animals must have had other purposes for play-type activities that conferred benefits through evolution. The not-play function of play states that simple locomotor play as exhibited by reptiles is about survival; being agile and adept on their legs helps them get away from predators more rapidly than less adept animals or helps them get to their own food faster. However, locomotor play is also good for physical wellbeing, contributing to muscular growth, flexibility/agility, and coordination; all activities shown in playful activity. The not-play function of play as exhibited by social mammals, such as monkeys, apes, dogs and meerkats, is connection: individuals may not “like” each other, but when the big cat comes around, they all stick together to chase it away or minimise loss in the group. Social play also helps with social wellbeing, belongingness, social connections, and acceptance. Additionally, the not-play function of emerging neural networks is resilience, problem solving, emotional regulation and flexibility. Therefore, it is possible to participate in playful-type activities without them being playful—self-chosen, engaging and satisfying; this may be one of the reasons that adolescents choose not to participate in sport and games as they move into secondary or high school.

In an effort to address reducing levels of activity, the “Girls on the Move” programme in Scotland set up community-based projects with the aim of improving girls and young women’s (aged from 11 to 18 years) engagement in physical activity, building on the desire for activity with peer groups and away from other distractions. The programme was reported in 2013 and showed that six-out-of-ten girls maintained their involvement in the projects and that four-out-of-ten girls had high levels of attendance. However, the conclusion of the report was that, while such short-term community-based projects can contribute to daily activity, organised sessions need to be supplemented with other forms of physical activity (e.g., physical education, active living) if girls and young women are to attain the U.K. government recommended 60 min of moderate-to-vigorous physical activity per day [39].

## 10. Play Deprivation

If it is recognized that play is good for making connections in a child’s brain, good for muscular growth and coordination, for socialisation, making friends and cognitive development and creativity [34]; then, not playing or being deprived of play opportunities would have a detrimental effect on the child or young person. The effects of play deprivation are complex, as there are many factors that affect the growing child, and there are always exceptions to the rule: children who will play and thrive in extreme conditions; however, the three main causes of play deprivation are the child, the environment and others (usually adults).

It may seem strange that children limit their own play, but looking at the characteristics of play, being in a sufficiently safe place, physically and psychologically, is a necessary factor [15]. Children under stress will do what they can to survive before they begin to play; if the stress factors continue long enough, the child experiences severe play deprivation. Most self-imposed play deprivation will come from feelings of fear or insecurity prompted by others or by the environment, whether those concerns are real or imagined. Though rare, there are instances in the modern world of carers who lock up their children, isolating them from contact with others and play spaces; the child’s response on being freed is to seek nourishment and other basic needs for survival rather than play. Work with animals has shown that if the play deprivation is short-lived, the animals bounce back and play more, almost as if they were trying to catch up; however, if the deprivation goes on too long, then brain structures and systems will be affected [40,41].

The extreme effects of adults’ actions on children may be seen in the extended play deprivation experienced by a large group of Romanian orphans that came to the attention of a wider community after the fall of the Communist government in 1989. As a result of government policies, the number of orphans rose during the regime, while the support to orphanages fell as a result of economic savings. After the fall of the regime it was discovered that some children were treated as subhuman by several of their carers and had been tied to their cots for the majority of their early childhood, unable to interact with others.

In a small-scale study measuring the brains of Romanian orphans who had been maltreated in their early years, Chugani *et al.* found that the orphans were “metabolically less active” in parts of the brain, especially the inner limbic area, linked to recognition of faces and emotions, two crucial components of bonding and attachment [42].

Some of the children were adopted by families from the USA and U.K. and were the subject of later research. When Beckett *et al.* [43] reported on the progress of the Romanian adoptees in the USA (compared to English adoptees), they found that the early effects of institutionalized care had lasting effects on the cognitive ability of the children, though there was good improvement shown by some of the most affected after intervention. This finding was consistent with the outcomes of the Therapeutic Playwork project [44], which did intense playwork with orphans in Romania and found that some of the children who were limited in size (compared to active peers) and considered by their carers to be brain damaged were able to respond positively when offered a stimulus and appropriate play cues [45].

The effects of play deprivation caused by the environment have already been touched on, though it follows that if an environment is impoverished (*i.e.*, lacking balanced stimulation opportunities), then children and young people will not be able to play to the extent of their abilities and desires. Additionally, the longer this environmental deprivation goes on, the more “normal” it may be considered by the adults and children who use that environment. The simple fact is that children need to be able to exercise their bodies, minds and emotions, exploring varied environments that give them access to others in those environments. While it is true that children and young people will play in the spaces they are in, the more stimulating those spaces are, the more they will be able to play.

The arguments for play and the explanations of not-play and play deprivation show how “play is critically important to human development and evolution” [33]; the drive to play while serving physiological, biological ends also contributes to children and young peoples’ mastery of the environment and their bodies and their development of higher level thinking and imagination. The loss of play opportunities in children and young people’s lives is potentially having a more profound impact on them than being overweight, as important as the ramifications of that may be on them and society.

## 11. Technology and Play

The impact of technology has affected humans for the last 200 years, but has escalated dramatically in the last thirty with the increase in available energy and computer-based technology; how this is affecting children’s play in all quadrants needs to be fully explored.

Play is what children get up to in their own lives, in their own way, every day. There is no agenda to what they do; they do it if they find it engaging and satisfying. The opportunities to play in the twenty-first century are quite varied from earlier generations, but carry the same interests for children; finding out about each other, exploring the world and beginning to make a difference in it. Yet, there has been an exponential growth in access to technology based activity and “play” in recent years. In addition to playing at home and in the street, children and young people may play everywhere in the real and virtual worlds; they will be healthier if they have the chance to balance these activities in their daily lives. As well as technology facilitating games, such as Neknomination, there is growing concern that violent video games leave teenagers “morally immature” [46] with weakened empathy for others; and so, perhaps, one of the reasons why drinking games are seen as playful and “fun” rather than dangerous.

Canadian-based research [47] working with a small cohort carried out an in-depth exploration of teenagers’ behaviour playing video games. More than half of the gamers were found to play video games between one and three hours every day, with violent games the most common activity; “violent” games were defined as those where players acted out the killing, decapitating or mutilating of other human characters. Concerns arose with teenagers who spent more than three hours every day in front of a screen, continuously playing violent games without any other real-life interaction. Empathy, trust and concern for others were found to be delayed in those who played these games excessively. The adult research team then suggested that a possible solution was for parents to place the teenagers in social situations, such as working for charities, where they get to see other people’s perspectives or needs. With all of the evidence of intrinsic motivation and self-chosen activity being critical to positive engagement, it may soon be considered impractical to try to overcome biological and culture motivators for adolescents’ behaviour with such adult-directed programmes.

## 12. Adult Roles and Attitudes

In recognising the effects of play deprivation and the benefits of self-chosen and engaging play, adults should do all that is possible to help children and young people access a variety of environments, with natural and unpredictable elements. These spaces should feel safe, psychologically and physically, for children and young people, and adults should understand the value and benefit, so that they encourage more play and experimentation. In the twenty-first century, too many children are being denied stimulating places to play because of the changes in the environment away from bio-diverse landscapes into designed spaces or indoor provision (with computers, videos and multi-media) that only meet limited interests. This is because too many adults believe that such “modern” environments are preferable to less comfortable ones without access to constant heat, hot water and every modern gadget. Children and young people will do what they have done for millennia; they will play, thrive and adapt in the environments in which they find themselves. It is adults who control those environments, and who try to control children and young people, who need to change. “For all sorts of reasons our society has restricted children’s and young people’s play; to remove restrictions and reverse a potentially damaging trend requires a change in attitudes across adult society” [48].

## 13. Summary

This paper has explored the motivations, personal, biological and cultural, that impact on teenagers’ play and choice of playful pastimes. It has shown that humans and particularly teenagers, due to the changes they experience in adolescence, need a multiplicity of varied stimuli and the opportunity to engage with them for extended periods from an early age; six-month programmes, while showing progress, are not enough to change underlying behaviours. Ideally, these opportunities should be a balance carried out with peers and supported by adults with an understanding of the drivers motivating young people. Teenagers continue playing beyond early childhood, though the play activities change and meet new needs. While pastimes, like Neknominate, parkour and buildering, are not recommended activities for teenagers, they may be understood as a response to adolescence and a desire for deep play, denied in other parts of the teenagers’ lives through the well-intentioned acts of parents and other adults. As reported, attitudes across adult society need to change, just as adults have radically changed the econiches that children and young people find themselves playing in throughout recent decades. The biological and cultural influences need to be considered and balanced, with the roles of policy makers and media reporters given particular attention alongside the impact of new technology.
